# A Spherical Phase Space Partitioning Based Symbolic Time Series Analysis (SPSP—STSA) for Emotion Recognition Using EEG Signals

**DOI:** 10.3389/fnhum.2022.936393

**Published:** 2022-06-29

**Authors:** Hoda Tavakkoli, Ali Motie Nasrabadi

**Affiliations:** Department of Biomedical Engineering, Faculty of Engineering, Shahed University, Tehran, Iran

**Keywords:** emotion recognition, subject-independent classification systems, brain-computer interface, nonlinear dynamic analysis, symbolic time series analysis, phase space partitioning, cosine similarity

## Abstract

Emotion recognition systems have been of interest to researchers for a long time. Improvement of brain-computer interface systems currently makes EEG-based emotion recognition more attractive. These systems try to develop strategies that are capable of recognizing emotions automatically. There are many approaches due to different features extractions methods for analyzing the EEG signals. Still, Since the brain is supposed to be a nonlinear dynamic system, it seems a nonlinear dynamic analysis tool may yield more convenient results. A novel approach in Symbolic Time Series Analysis (STSA) for signal phase space partitioning and symbol sequence generating is introduced in this study. Symbolic sequences have been produced by means of spherical partitioning of phase space; then, they have been compared and classified based on the maximum value of a similarity index. Obtaining the automatic independent emotion recognition EEG-based system has always been discussed because of the subject-dependent content of emotion. Here we introduce a subject-independent protocol to solve the generalization problem. To prove our method’s effectiveness, we used the DEAP dataset, and we reached an accuracy of 98.44% for classifying happiness from sadness (two- emotion groups). It was 93.75% for three (happiness, sadness, and joy), 89.06% for four (happiness, sadness, joy, and terrible), and 85% for five emotional groups (happiness, sadness, joy, terrible and mellow). According to these results, it is evident that our subject-independent method is more accurate rather than many other methods in different studies. In addition, a subject-independent method has been proposed in this study, which is not considered in most of the studies in this field.

## Introduction

Emotions, which refer to a psychophysiological process resulting from understanding an object or situation, affect our daily lives by directing actions and moderating motivation. Most human works are influenced by emotions, from how we think to decision making and our behavior or communication. Positive emotions enhance human health and work effectiveness, whereas negative emotions probably pave the way for health issues. For example, the World Health Organization (WHO) had predicted that depression would be the most common disease in the world by 2020 (Byun et al., 2019), and untreated depression increases the mortality rate and may cause suicidal behavior, which is a severe public health problem (Franklin et al., 2017). Moreover, it was confirmed that learning processes are deeply affected by emotional intelligence, especially for information extraction, in which its importance is most apparent (Salovey and Mayer, 1990; Goleman, 1995).

In recent years, many different uses of Human-Computer Interaction (HCI) systems have become commonplace (Chai et al., 2016), and one of the applications of HCI systems that have been recently taken into consideration are systems that need to detect and analyze the emotions, such as rehabilitation systems or health care, computer video games, etc. Designing such systems requires comprehending and recognizing emotions (Stickel et al., 2009; Bajaj and Pachori, 2015; Verma and Tiwary, 2017), so understanding the user’s emotional state is assumed as a significant factor. In the past decade, emotion recognition researches from different modalities [e.g., physiological signals (Khezri et al., 2015; Yin et al., 2017), facial expression (Cohen et al., 2000; Ioannou et al., 2005; Flynn et al., 2020; Proverbio et al., 2020), etc.] have been grown up and recently, thanks to the existence of cost-effective devices for capturing brain signals [electroencephalographic (EEG) signal] as input for systems that decode the relationship between emotions and electroencephalographic (EEG) variation, researchers in the field of Brain-Computer Interface (BCI) have been studying emotion recognition to make affective BCI (aBCI) systems (Mühl et al., 2011; Ju et al., 2020; Torres et al., 2020).

Emotion recognition is the research area trying to design systems and devices capable of identifying, interpreting, and processing human emotions, which would lead to the probable creation of machines capable of interacting with emotions. Emotional states play an essential role in decision-making or problem-solving, and emotional self-awareness can help people manage their mental health and optimize their work performance. Some researchers believe that using of EEG-based BCI in emotion recognition systems will soon increase, and they could be used for emotion recognition in daily life for several purposes, such as gaming and entertainment, health care facilities, teaching-learning scenarios, and optimizing performance in the workplace or some other applications (Torres et al., 2020). Therefore, the study of emotion recognition seems to be very practical. One of the most important challenges in this field is to decode the EEG’s information and map it to specific emotions, which we intend to address in this study. In this regard, several investigations have been conducted using various procedures in the past, which we will review in the next section.

**Figure 1:**
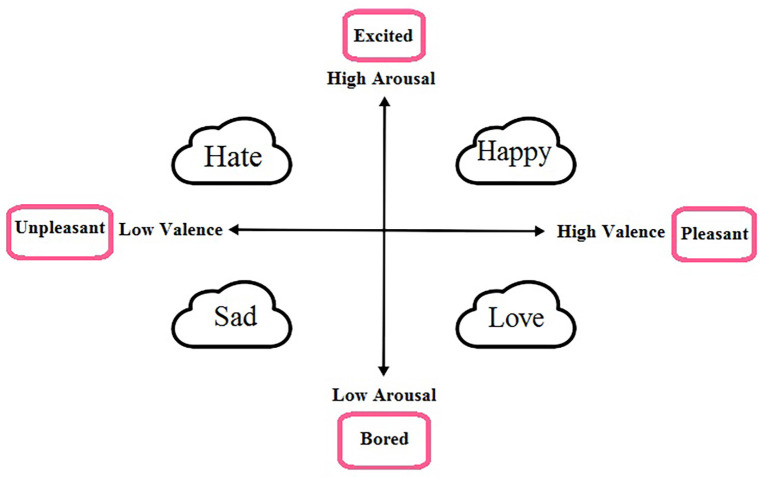
Representation of dimensional model for emotions , this model defines all emotions on a two dimensional space, valence and arousal. Valence denotes the polarity of emotions [positive (pleasant) or negative (unpleasant)] and arousal shows the amount of excitement [high (excited) or low (bored)]. As seen in the figure, discrete emotions, based on the amount of arousal and valence values, can be shown on this dimensional model.

Explaining a person’s emotional state is one of the main issues in emotion recognition. Dimensional and discrete models are two models for describing emotional states. In general, some fundamental emotional states, such as sadness, happiness, hate, joy, surprise, terrible, and anger, express discrete models (Ekman, 1992), whereas the valence-arousal space represents the dimensional model (Mehrabian, 1996). The dimensional model describes all emotional states using the valence and arousal axis. The range of qualitative changes varies from negative to positive, and each one is interpreted in a way. While high to low-level activity in the arousal state represents exited to boring conditions, the positive and negative valence score states demonstrate pleasant (e.g., happiness) to unpleasant feelings (e.g., sadness). Figure 1 illustrates the arousal-valence dimensional model and some of the discrete emotions.

In recent years, emotion recognition has received much attention in many fields of science. Several approaches have been introduced to identify or classify emotions, which will be reviewed below.

Several approaches have been used for emotion recognition in the literature. Since a person’s emotional state has an external appearance on his/her face and the emotions can be recognized from his/her face, one of the most common approaches to recognize emotions is the facial expression recognition systems (Koelstra et al., 2012; Sharma et al., 2019; Yadav, 2021). Another common approach to recognizing emotions is speech analysis done in a wide range of studies (Schuller et al., 2003; Jahangir et al., 2021). Despite the positive results reported from these methods, the person may want to hide his/her inner feelings. Also, these recognition methods have a critical limitation because they are dependent on the cultural and social environment of the subjects. This limitation may be overcome by using physiological signals such as electromyogram (EMG), electroencephalogram (EEG), skin temperature, blood volume pulse, etc. (Yoon and Chung, 2013). Thus, the emotion recognition systems moved towards processing physiological signals because they are more accurate due to non-being controllable by the subject. Physiological changes are the basis of emotions in our body (Alhagry et al., 2017), so analyzing these signals, like electrocardiogram (ECG), electromyogram (EMG), galvanic skin response (GSR), blood volume pressure (BVP), and electroencephalogram (EEG) make emotions to be recognized (Koelstra et al., 2012; Moharreri et al., 2018; Bao et al., 2021; Ebrahimzadeh et al., 2021a). Because the source of a person’s emotions is the central nervous system (CNS), the brain signals seem to be the most appropriate option for extracting emotional information. The most common signal that shows the brain’s electrical activity is the electroencephalogram (EEG), which is widely used in extracting and analyzing brain system information due to its non-invasiveness, easy recording, and very high temporal resolution (Ebrahimzadeh et al., 2019a, 2021b; Zhong et al., 2020; Sadjadi et al., 2021). The EEG signal actually measures the brain’s activity, which is responsible for regulating and controlling emotions (Soroush et al., 2020), so emotion recognition systems based on EEG signals have been favored by researchers (Takahashi, 2004; Bos, 2006; Petrantonakis and Hadjileontiadis, 2011; Bajaj and Pachori, 2015; Pham et al., 2015; Singh and Singh, 2017).

There are several linear feature extraction techniques, including time and frequency domain methods (Taran and Bajaj, 2019) and a variety of traditional machine learning methods such as Support Vector Machines (SVM), Linear Discriminant Analysis (LDA), Artificial Neural Networks (ANN) and functional/effective connectivity (Zhang et al., 2020; Ebrahimzadeh et al., 2021c; Seraji et al., 2021) for EEG-based Emotion Recognition systems (Zhong et al., 2020; Bao et al., 2021). Statistical features of EEG signals, such as mean value, power of the signal, and the first and second difference, are usually used as time-domain features (Takahashi and Tsukaguchi, 2003), and frequency domain characteristics like the power spectrum of each EEG different bands are used as frequency domain features (Wang et al., 2011). The above methods are all linear analysis methods. But the EEG signal is generated by a very complex system (i.e., the brain) that is supposed to have a nonlinear, non-stationary, and chaotic behavior (Soroush et al., 2020). So, it is better to adopt a suitable non-linear method to extract information from this nonlinear complex system.

Regarding the nonlinear nature of EEG (Stam, 2005), it seems nonlinear features and tools outperform emotion recognition. Non-linear features, such as Fractal dimension (FD; Sourina and Liu, 2011; Liu and Sourina, 2014), sample entropy (Jie et al., 2014; Raeisi et al., 2020), and nonstationary index (Kroupi et al., 2011), have been used in mentioned studies to recognize emotions. Among the nonlinear approaches that have been performed, some of them require advanced combinations of a large number of features or use complicated systems or algorithms, so their computational cost is high, and also the clinical meaning of each variable is fully blurred within complex classifiers (García-Martínez et al., 2017; Ebrahimzadeh et al., 2019b), so, in this study, we tried to propose a method to overcome this issue that has a low computational cost.

One of the nonlinear analysis tools is Symbolic Time Series Analysis (STSA), which has been favored over the last few years in many research areas, including mechanical systems, artificial intelligence, data mining, and the bio-signal processing (Alcaraz, 2018). One of the main advantages of symbols is the effectiveness of numerical computation that could be considerably boosted relative to what is achievable by directly analyzing the original time series (Daw et al., 2003). This is an essential feature in utilizing systems with limited computational speed and memory capacity used in real-time mobile platform applications. Additionally, analysis of symbolic data is typically robust to the measurement noise (Daw et al., 2003; Reinbold et al., 2021). There, to have low-cost and relatively simple devices, symbolization can be directly performed in the instrumentation software (Chin et al., 2005). Also, it has been shown that, in the case of noisy signals, symbolization can boost the signal-to-noise ratio (Daw et al., 2003). Symbolic analysis has been used to investigate many biological systems features (Daw et al., 2003; Glass and Siegelmann, 2010; Schulz and Voss, 2017; Awan et al., 2018), in between, neural pathologies diagnosing (Lehnertz and Dickten, 2015) and neural systems laboratory measurement are the most notable candidates. For example, Azarnoosh et al. (2011) used symbolic analysis methods to determine mental fatigue.

The original data must be discretized into a matching sequence of symbols to perform this technique. The transformed version of the original data contains temporal information of the original signal. So, an important stage in STSA is the **data partitioning** for **generating the symbol sequence** (Daw et al., 2003). Traditional methods for generating the symbols and selecting the location of partitions have used the mean, midpoint, or median of the data. Other methods that have been suggested are: to make equal size intervals over the data range (“Uniform Partitioning”; Rajagopalan and Ray, 2006) or equal probability regions over the data range (“Maximum Entropy partitioning”; Rajagopalan and Ray, 2006). Tang et al. (1997) argued that a mean-based binary symbol set partitioning could be used for dynamics reconstruction of nonlinear models, even upon noisy dynamics. Uniform partitioning of EEG signals was applied to identify precursors to seizures by Hively et al. (2000). There are also other approaches to defining symbols, including “symbolic false nearest neighbors partitioning (SFNNP)” (Buhl and Kennel, 2005) and “wavelet-space partitioning (WSP)” (Rajagopalan and Ray, 2006).

One of the essential tools in nonlinear system analysis is the phase space, which has important information about the system (Soroush et al., 2020). Relying on the precious knowledge of the phase space, in the present study, we try to extract this information by the symbolic analysis of this space to find a general pattern for emotions and classify some emotional states based on these patterns.

Another problem with most methods used to recognize or classify emotions is that they are subject-dependent. Because the people’s opinions towards feelings are subjective (Soroush et al., 2020), being dependent on the person is a weakness for an emotion recognition system, and designing a system independent of the individual, which can achieve a general pattern for each emotion, is a significant advantage to it counts. This study uses the leave-one-subject-out validation method to provide a subject-independent system for emotion recognition.

The theoretical framework of a novel STSA-based approach has been introduced in the present study to recognize the dynamical patterns and its experimental validation for emotion recognition using EEG signals. Considering the advantages mentioned for the symbolic time series analysis method in the analysis of signals and nonlinear systems (such as reducing the sensitivity to noise, decreasing the numerical computation, etc.) and according to the complex and non-linear nature of the EEG signal, our goal in this study is to develop a new method based on the symbolic time series analysis approach, which uses the most constructive information from the phase space with the least computational cost. To extract the information from the whole brain system, all 32 EEG channels would be utilized to construct the phase space. This, however, would dramatically increase the dimensionality and computational demands. We propose Spherical Phase Space Partitioning (SPSP) to overcome this issue, which uses for partitioning the phase space, generating the symbolic sequences, and achieving a general index pattern for each emotional state. Then, the symbolic sequences are compared and classified based on the maximum value of a similarity index. A subject-independent protocol will be used to enhance generalization ability.

So, this study aims to improve the precision of emotion recognition systems with a simple, low computational cost and subject-independent algorithm. The remainder of the manuscript is organized as follows. “Materials and Methods” explains the database put to analysis in the study, provides a brief description of the traditional STSA and cosine similarity index methods, and finally introduces the proposed method for symbolization. The results are given in “Results” Section and then discussed in “Discussion” Section. Ultimately, the most important conclusions are brought to light in the final part of “Discussion” Section.

## Materials and Methods

### Dataset

DEAP database was applied in the current study, a multimodal emotional database for human emotions analysis, taken from subjects who watched music videos (Koelstra et al., 2012). The DEAP database consists of EEG and peripheral physiological signals of 32 subjects, in which 40 music videos were displayed for each subject. EEG and peripheral signals were recorded at a sampling rate of 512 Hz. In this study, pre-processed data provided by DEAP authors were used. After down-sampling the EEG data to 128 Hz, they were averaged to the common reference. Afterward, eye artifacts were taken away, and a high-pass filter was used. The duration of each video was 1 min, and after each movie, subjects scored the movie in each of the five dimensions of a self-assessment questionnaire. Emotional response covers five dimensions: dominance, valence, arousal, liking, and familiarity. Moreover, some videos have featured discrete labels like happiness and sadness. This study is based on the discrete model for emotion, using five emotional states sadness, happiness, hate, joy, and mellow.

### Symbolic Time Series Analysis

Symbolic Analysis of a signal is a new approach in which continuous signals are converted to symbol sequences using partitioning of the continuous signal domain (Srivastav, 2014). This method is a special case of symbolic dynamics, which is used as its substitution, especially for signals, because of the inherent limitations in the symbolic dynamics method for executing for signals (the symbolic dynamics approach is often applied to systems), particularly in the presence of noise (Daw et al., 2003).

According to the concept of symbolic dynamics, it would be possible to describe the features of a dynamical system by partitioning its phase space into *K* sets that are mutually disjoint {S_1_, S_2_,…, S_K_}, so each possible trajectory transforms into a sequence of symbols (Robinson, 1998). Typically, a generating partition is needed for applying the concepts of symbolic dynamics, which match an **exclusive** assignment of symbolic sequences to every system’s trajectory. It should be noted that this requirement is usually ignored in real-world applications because of noise; yet, even if there is no noise, generating partitions are either not available or could not be estimated (Hirata et al., 2004). Hence, as an alternative to symbolic dynamic, performing Symbolic Time Series Analysis (STSA) has been favored recently in many applications (Donner et al., 2008).

**Figure 2:**
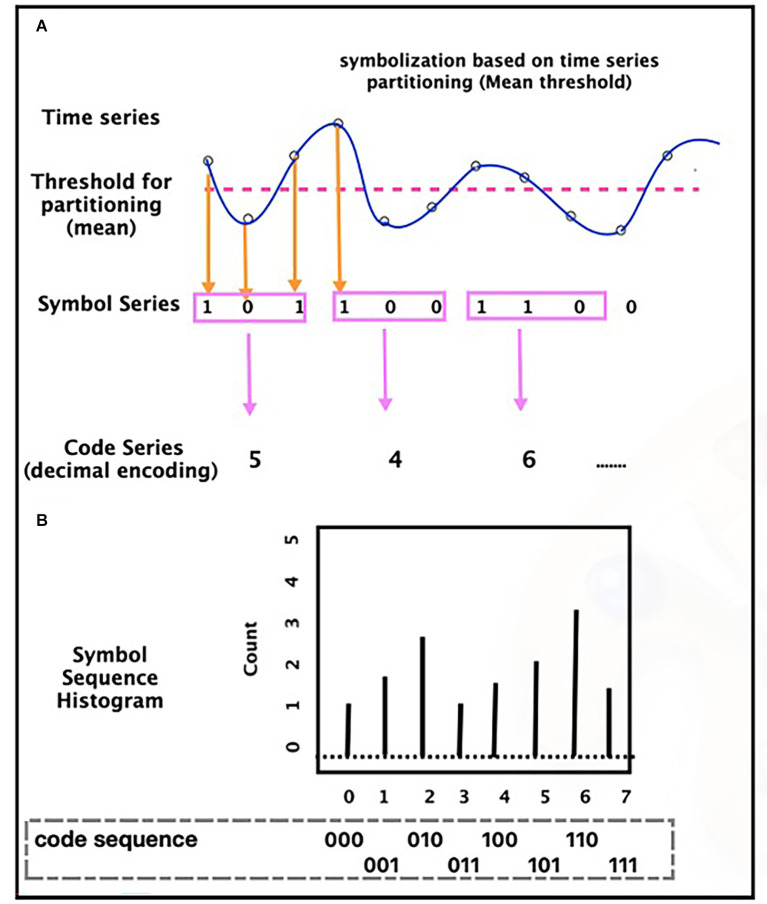
Time series symbolization process **(A)** and tabulating a histogram of symbol sequence **(B)**—based on a threshold (for example, **mean**, in this figure), the time-series convert to a symbol sequence (series). Based on the length considered for the words, the sequence of symbols becomes a series of words. Then, the analysis of the words is usually performed by statistical analysis of the words occurrence, frequency, like histogram.

In traditional symbolic dynamic (or symbolic time series analysis), the next step after symbolization is symbolic sequence (**words** in the symbolic dynamics letter) construction which is made by gathering groups of symbols together to identify temporal patterns. Finally, the analysis of these symbol sequences (words) is usually performed by statistical analysis of the word occurrence frequency in the symbolic sequences. Figure 2 illustrates this process for a time series.

As mentioned in section 1, there are some criteria for **partitioning** and discretizing the signal, such as mean, median, mean ± variance, and some other new approaches like False Nearest Neighbor Partitioning (Kennel and Buhl, 2003), Wavelet Partitioning (Rajagopalan and Ray, 2006) and Hilbert–Hung Partitioning (Sarkar et al., 2009). This article aims to introduce a new approach for partitioning the continuous data in the phase space based on the **spherical partitioning**, which will be explained in Section “Proposed STSA-Based Emotion Recognition Method”.

### Cosine Similarity

A similarity measure is an important tool for determining the degree of similarity between two objects. It is believed that similarity measures are advantageous in pattern recognition, image processing, and machine learning (Ye, 2011). The cosine similarity measure is one of these measures, a widely used metric that is both a simple and effective (Xia et al., 2015). It is defined as the cosine of the angle between the two vectors, determines whether two vectors are pointing in roughly the same direction (Salton and McGill, 1986), and is obtained by dividing the inner product of two vectors by the product of their lengths.

This classic measure is applied for information extraction and is the most useful described measure for proving vector similarity (Salton and McGill, 1986). The formulate of the cosine similarity is simply as follows:


(1) $$similarity=\cos\theta =\frac{A.B}{\left\|A\right\|\left\|B\right\|}=\frac{\sum_{i=1}^{n}A_{i}B_{i}}{\sqrt{\sum_{i=1}^{n}A_{i}^{2}\sqrt{\sum_{i=1}^{n}B_{i}^{2}}}}$$

Where A_*i*_ and B_*i*_ are components of vectors A and B, respectively and *||x||* indicate the absolute value of the desired vector. As can be seen, the cosine similarity is obtained by dividing the inner product of two vectors by their lengths. This division eliminates the effect of the “length” or “magnitude” of the vectors, which is an important feature of cosine similarity. This lack of sensitivity to vectors’ magnitude is essential, especially in our study where we work with “codes” or “symbols”, instead of “amplitude” or “real values”. The lack of dependence on the domain seems very necessary and valuable. So we use this measure to find similarities between each emotional symbolic sequence and each emotion index, for classification purposes.

### Proposed STSA-Based Emotion Recognition Method

The adopted procedure of STSA to recognize emotions used in this study is explained briefly in this section. The adjusted STSA emotion recognition method uses the vector information generated by phase space partitioning, wherein the time-series data grow. The steps are as follows:

• Partitioning of the signal’s phase space and transforming time-series data from the continuous domain to the symbolic domain.
• Calculation of the similarity between each emotional state vector and reference (index) vectors for the classification of each emotional test vector.

#### Spherical Phase Space Partitioning Based Symbolic Time Series Analysis (SPSP–STSA)

**Figure 3:**
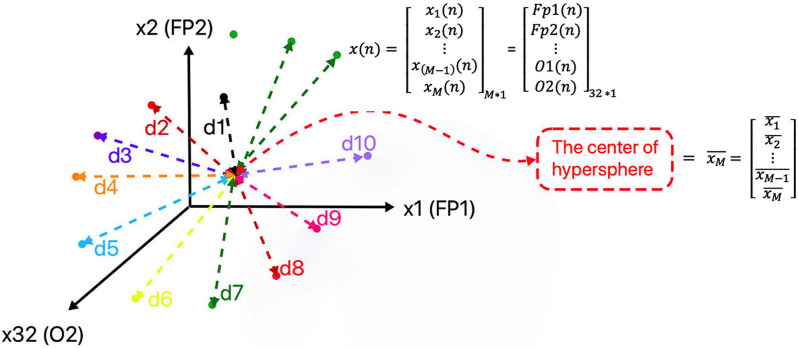
Representation of points and the distances of points from the center of the hypersphere, in the constructed phase space of the signal (due to the limitation of representation of space dimensions number, only three dimensions of phase space are displayed).


Because of the advantages of phase space in representing the nonlinear features of a system, partitioning the phase space was employed in this study. To analyze a set of signals, using partitioning the phase space, we first need to construct its phase space trajectory. All 32 EEG channels (Fp1, Fp2, AF3, AF4, Fz, F3, F4, F7, F8, FC1, FC2, FC5, FC6, Cz, C3, C4, T7, T8, CP1, CP2, CP5, CP6, Pz, P3, P4, P7, P8, PO3, PO4, Oz, O1, O2) is used to construct the phase space and each sample point “n” in the phase space, is constructed as follow:


(2) $$x(n)={\left[\begin{array}{c} x_{1}(n)\\ x_{2}(n)\\ \vdots \\ x_{\left(M-1\right)}(n)\\ x_{M}(n) \end{array}\right]}_{M\;*\;1}={\left[\begin{array}{c} Fp1(n)\\ Fp2(n)\\ \vdots \\ O1(n)\\ O2(n) \end{array}\right]}_{32\;*\;1}$$

where *n* = 1:N denotes the samples, *N* = 8,064 is the total points of each signal, *M* = 32 is the number of channels (the dimensional of phase space), x_i_(n) denotes the ith dimension of the space at the sample “n” and x(n) denotes each M dimensional point of the phase space (Figure 3). The matrix of all trajectory points for one EEG signal (for example: for one movie of one person with the length of N points) in the phase space will be as follows:

(3) $$X=\left[x(1),x(2),...,x(N)\right]={\left[\begin{array}{c} x_{1}(1),x_{1}(2),...,x_{1}(N)\\ x_{2}(1),x_{2}(2),...,x_{2}(N)\\ \vdots \\ x_{\left(M-1\right)}(1),...,x_{\left(M-1\right)}(N)\\ x_{M}(1),x_{M}(2),...,x_{M}(N) \end{array}\right]}_{M*N}$$

where X is an M*N matrix that indicates the total points of a trajectory for one EEG signal.

Then we use the new spherical partitioning idea to discretize the phase space and make symbols.

In the partitioning scheme, there are two main approaches:

-Uniform Partitioning

-Maximum Entropy Partitioning (ME)

Firstly, the maximum and minimum of the distances from the mean point are assessed, and equal-sized regions are obtained by partitioning the range between the maximum and minimum. These regions are mutually disjointed. A symbol from the alphabet (the set of symbols) then assigns each region. The data point is coded with a specified symbol if it is in a certain region. Therefore, a sequence of symbols is generated based on a given sequence of time series data (Uniform Partitioning).

Naturally, it seems to be more appropriate to partition regions due to their information, i.e., the higher the information, the finer the partitioning, and vice versa. To reach this goal, a partitioning method was applied so that the entropy of the generated symbol sequence is increased as much as possible (Rajagopalan and Ray, 2005). The process to obtain an ME partition is explained below.

**Figure 4:**
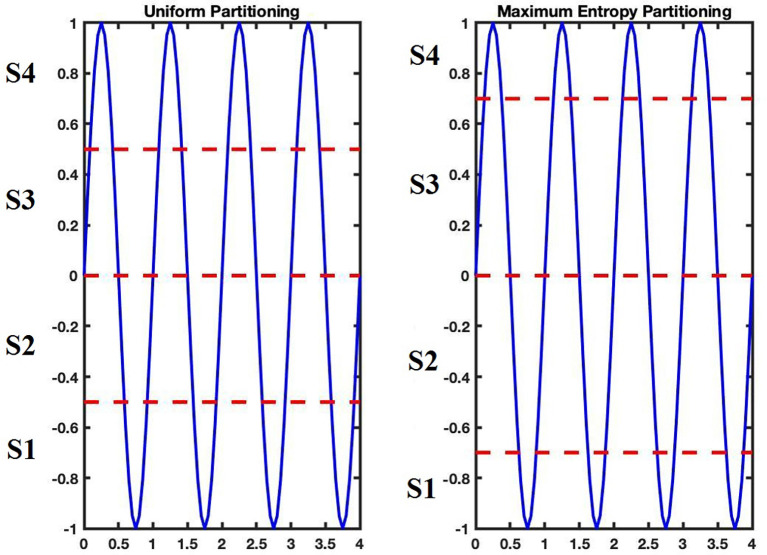
Uniform and Maximum Entropy (ME) partitioning examples with S = 4.

**Figure 5:**
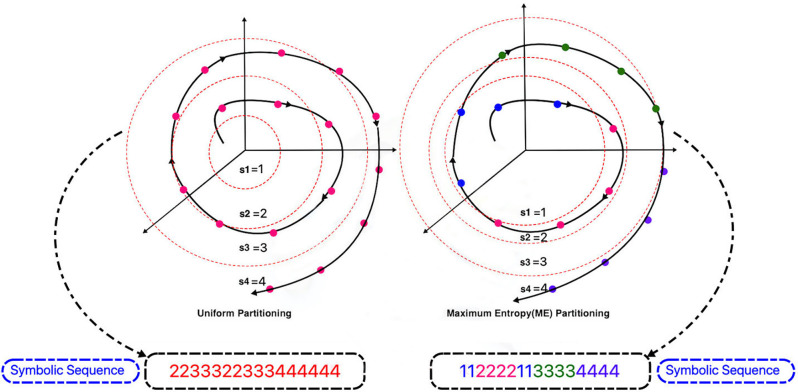
Comparison of uniform and maximum entropy partitioning in spherical partitioning and symbol sequence generation phase.

Suppose the length of the signal is L and the number of symbols is S (size of the alphabet). L samples of signals are set in ascending arrangement. A data segment of length [L/S] starts from the first sorted data point, makes a separate portion of the partitioning, in which *[x]* denotes the integral part of *x*. Figure 4 shows the difference between these two types of partitioning for a sine signal and Figure 5 shows this difference for a sample signal in three-dimensional phase space. The plot on the right in Figure 5 indicates ME partitioning for a sample signal, with *S* = 4. As predicted, the partitions’ size is not equal; nevertheless, the symbols have equal likelihoods. It is more probable that discrepancies in data patterns are revealed in ME partitioning than in other partitioning methods (Rajagopalan and Ray, 2006).

In our proposed spherical partitioning method, based on the ME partitioning approach, at first, each point’s distance (d) is calculated from the center of the hypersphere (Figure 3). Then the partitioning is done based on the number of symbols selected according to the rule described below.

The selection of symbol number **S** is essential in STSA and is an active research area. For instance, a small value of **S** might be insufficient to capture the features of the time series data. Besides, a large value of **S** could cause redundancy and unused computational resources. To select S, Rajagopalan and Ray (2006) applied an entropy-based approach, so we used this approach in this study. Presume H(k) signifies the symbol sequence Shannon Entropy which is acquired after k symbol partitioning:


(4) $$H(k)=-\sum_{i=1}^{i=k}p_{i}\log_{2}p_{i}$$

where *p*_i_ indicates the probability of occurrence of the symbol *s*_i_ and H(1) = 0.

If sufficient information content of the underlying data set, like the Maximum Entropy Partitioning situation, has been available, then H(k) would be log_2_(k). To indicate the change in entropy in terms of the number of symbols (**S**), we describe h(.) as follows:


(5) h (k)=H(k)−H(k−1)   ∀ k≥2

**Figure 6:**
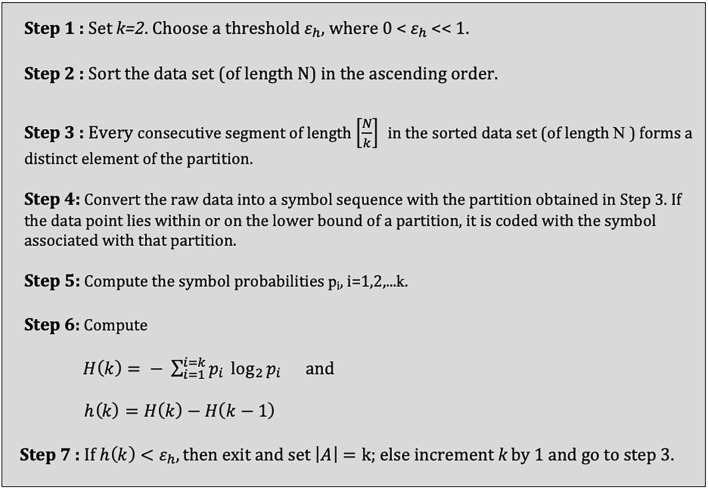
Algorithm for selecting the number of symbols.

A threshold *ε_h_* was defined, where 0 < *ε_h_* << 1 and start with k = 2; for each k, the symbol probabilities *p*_i_ (i = 1, 2, …, k) are computed, and H(k) and h(k) are calculated by equations (4) and (5) respectively and when h(k) < *ε_h_*, exit the algorithm and choose k as the number of symbols (**S**) (Rajagopalan and Ray, 2006; Figure 6).

After selecting the number of symbols, it is the time to partition the phase space, and in this study, a new approach was considered as Spherical Phase Space Partitioning (SPSP). Consider “M” EEG channels shown by *x*_*m*_(*n*) m = 1, …, M (m: counter of dimension, M: total number of state-space dimension (EEG channels)); n = 1, …., N (n: sample counter, N: length of time series). At first, the phase space is constructed using 32 channels. The mean point (center of hypersphere) is calculated as follows:


(6) $$\bar{x}=\frac{1}{n}\left(\sum_{i\;=\;1}^{n}x_{i}\right)\underset{\to }{in\;M-\dim ensional\;state\;space}\;\bar{x_{M}}=\left[\begin{array}{c} \bar{x_{1}}\\ \bar{x_{2}}\\ \vdots \\ \bar{x_{M-1}}\\ \bar{x_{M}} \end{array}\right]=\left[\begin{array}{c} \frac{1}{n}\left(\sum_{n\;=\;1}^{N}Fp1(n)\right)\\ \frac{1}{n}\left(\sum_{n\;=\;1}^{N}Fp2(n)\right)\\ \vdots \\ \frac{1}{n}\left(\sum_{n\;=\;1}^{N}O1(n)\right)\\ \frac{1}{n}\left(\sum_{n\;=\;1}^{N}O2(n)\right) \end{array}\right]$$

where $\bar{x_{M}}$ denotes the center of a hypersphere in M dimensional state space and $\bar{x_{\iota }}$ indicates the mean value of each dimension obtained by the average formula mentioned above. Then, based on the trajectory points distance from the center point (d) (Figure 3) and based on the ME approach, the phase space is partitioned to **S** symbols. As the trajectory evolves in the phase space, symbol sequences would be made (Figure 5).

#### Classification: Cosine Similarity

After partitioning the phase space and generating the symbol sequences, we need to compare each person’s emotional state symbolic vector with some emotional indexes and detect and classify each emotional state. We use cosine similarity for this purpose. For each subject, we calculated the cosine similarity of the symbolic sequence of that person with the index of each emotional group, which is obtained by averaging over the symbolic sequences of other subjects, and the classification of each test data is based on the maximum value of this criterion. The performances of these models were examined using the leave-one-subject-out cross-validation method.

**Figure 7:**
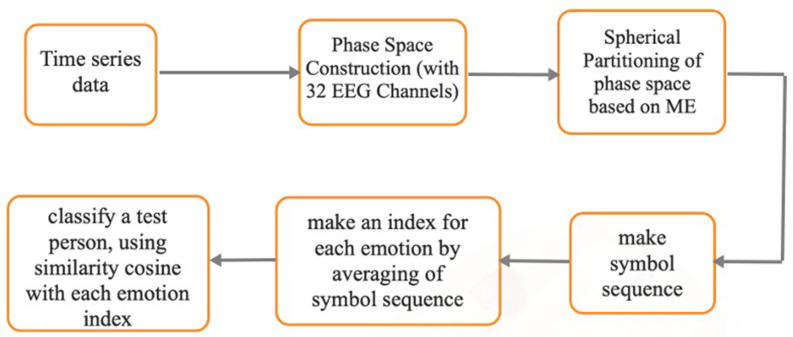
The block diagram of the proposed method.

The process of the proposed method is shown in Figure 7. Also, we are preparing the codes in a user-friendly way, and after preparing them, we will put them on the GitHub.

## Results

**Figure 8:**
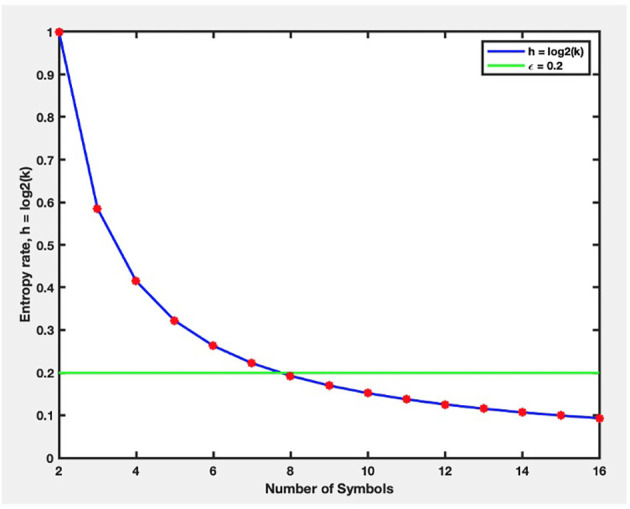
The changes in entropy (h) against the number of symbols (S). (h) monotonically was reduced with increasing the amount of S and becomes less than *ε_h_* when *S* = 8.

Our new proposed approach to STSA was applied to the openly accessible emotion dataset (the DEAP dataset; Koelstra et al., 2012). All 32 EEG channels have been used for phase-space construction. The phase space was constructed and partitioned individually for each person and each video. Selecting the number of symbols **S** was one of the critical phases in STSA, which was performed based on the entropy-based criteria described in “Proposed STSA-Based Emotion Recognition Method” Section. The threshold parameter *ε_h_* was selected to be 0.2. Figure 8 shows the change in entropy *h* (Equation 5) against the number of symbols **S**. As is shown, *h* monotonically was reduced with increasing the amount of **S** and becomes less than *ε_h_* when **S** = 8. Thus, the number of symbols **S** was selected to be 8.

After selecting the number of symbols, the phase space was constructed and partitioned for each person’s movie based on the ME Partitioning approach. As the system evolves through time, it moves through several blocks in its phase space, and the matching symbol *j* (*j* = 1, 2,…, 8) is allocated to it thus a data sequence is adapted to a symbol sequence s_i1_, s_i2_, s_i3_,…. Therefore, the symbol sequences represent coarse-graining of the trajectories time evolution. After all intended movies of all participants were symbolized, we used cosine similarity (Equation 1) to find the maximum similarity between each person’s symbolic emotional EEG signals and each emotional index for classification purposes.

**Figure 9:**
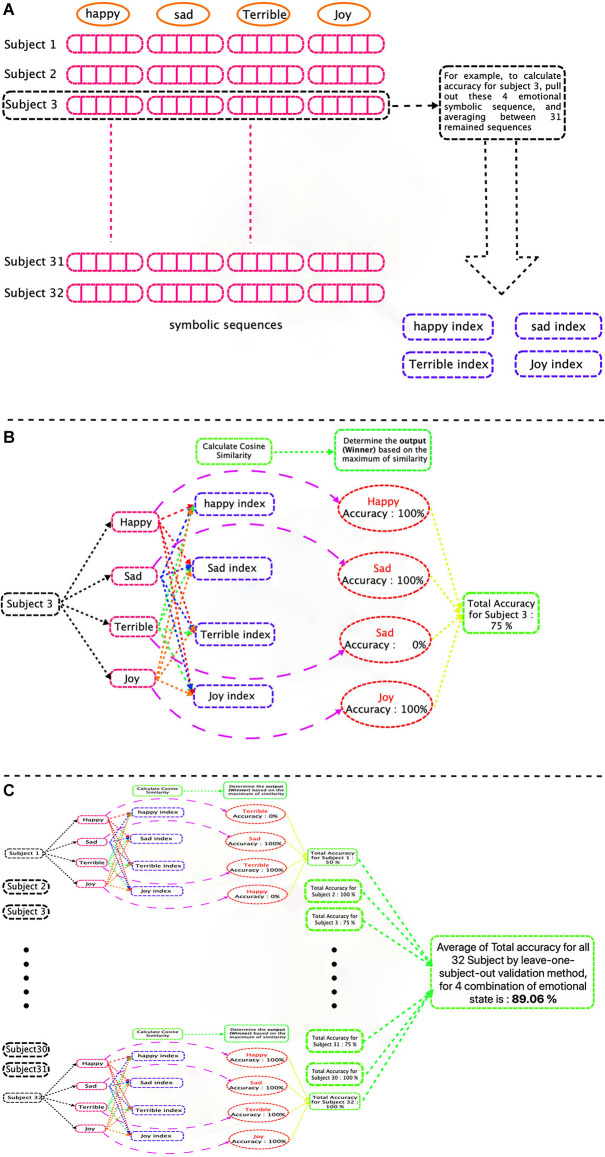
Graphical flowchart of classification process of a signal (four groups of emotions case). **(A)** Training phase and calculate emotional indices. **(B)** Test Phase. Each test subject’s EEG emotional symbolic sequence, is compared to four symbolic indexes (for each emotion) and based on the maximum value of the cosine similarity, is assigned to one emotional group and finally, the accuracy is calculated. **(C)** Calculate total accuracy for all subjects.

To validate the classification results, we used the leave-one-subject-out cross-validation method (i.e., to compute the accuracy for each subject, that person was excluded, and the average of all 31 remaining sequences was calculated as an index for each emotional state.) Then, the cosine similarity between each emotional index and a test vector was computed, the accuracy for each subject was computed, and the total accuracy was obtained by averaging on all subjects. This procedure is shown in Figures 9A–C.

The procedure was performed for four different combinations of emotions by adding one emotion in each step. At first, we started with two emotions, namely happiness, and sadness, and our proposed method was able to classify these two emotional groups with a precision of 98.44%. In the next step, we added Joy to the groups, and we were able to distinguish these three groups with an accuracy of 93.75%. For four groups classification, we were able to classify the emotions including happiness, sadness, joy, and terrible and separated four groups with an accuracy of 89.06% and finally, for five groups of emotions (happiness, sadness, joy, terrible, and mellow) the classification accuracy was 85%.

It should be noted that in all four combinations of emotional states, an increase in classification accuracy is observed compared to some previous studies. Our proposed method results in comparison with the other methods in the literature, on the same dataset or some other datasets, were shown in Tables s 1–4.

**Table 1:** Comparison of different studies for emotion recognition using EEG—two groups of emotions.

	Method description	Dataset	Emotions	Accuracy
Wang et al. (2014)	Power Spectrum Features, wavelet features, nonlinear dynamical features, SVM, six subjects	Personal Data	Positive and Negative	87.53%
Liu and Sourina (2013)	Higher Order Crossing, six statistical features, Fractal Dimension, SVM	Personal Data	—	87.02%
		DEAP		90.35%
Proposed Method	Symbolic Time Series Analysis, similarity index	DEAP	Happy and Sad	**98.44%**

*Bold value indicates high percentage of accuracy*.

**Table 2:** Comparison of different studies for emotion recognition using EEG—three groups of emotions.

	Method description	Dataset	Emotions	Accuracy
Brown et al. (2011)	Spectral Power Features, KNN, 11 subjects	Personal Data	Positive, Negative, and Neutral	85%
Liu and Sourina (2013)	Higher Order Crossing, six statistical features, Fractal Dimension, SVM	Personal Data	—	74.44%
		DEAP		84.41%
Proposed Method	Symbolic Time Series Analysis, similarity index	DEAP	Happy and Sad	**93.75%**

*Bold value indicates high percentage of accuracy*.

**Table 3:** Comparison of different studies for emotion recognition using EEG—four groups of emotions.

	Method description	Dataset	Emotions	Accuracy
Candra et al. (2015)	Wavelet Energy, Wavelet Entropy, SVM	DEAP	Happy, sad, angry, and relaxed	77.4%
Lin et al. (2010)	Power Spectral Density and asymmetry features of five frequency bands, SVM, 26 subjects	Personal Data	Joy, anger, sadness, and pleasure	82.29%
Liu and Sourina (2013)	Higher Order Crossing, six statistical features, Fractal Dimension, SVM	Personal Data	—	67.08%
		DEAP		80%
Proposed Method	Symbolic Time Series Analysis, similarity index	DEAP	Happy, sad, joy, and terrible	**89.06%**

*Bold value indicates high percentage of accuracy*.

**Table 4:** Comparison of different studies for emotion recognition using EEG—five groups of emotions.

	Method description	Dataset	Emotions	Accuracy
Jenke et al. (2014)	Higher Order Crossing, Higher Order Spectra, and Hilbert -Hung Spectrum features, 16 subjects	Personal Data	Happy, sad, angry, quiet, and curious	36.8%
Liu and Sourina (2013)	Higher Order Crossing, six statistical features, Fractal Dimension, SVM	Personal Data	—	61.67%
		DEAP		76.53%
Proposed Method	Symbolic Time Series Analysis, similarity index	DEAP	Happy, sad, joy, terrible, and mellow	**85%**

*Bold value indicates high percentage of accuracy*.

The classification accuracy is reported in Tables s 1–4, but in order to determine which emotions are easiest and which are the most difficult to distinguish, investigating the confusion matrices can be useful. The confusion matices for each groups of emotional state are shown in Tables s 5–8. Tables s 6, 7 show, for example, that joy is the most difficult emotional state to classify and has the highest rate of misclassification among the three and four emotional states. In addition, other emotional states can be analyzed using Tables s 5–8.

**Table 5:** Confusion matrix for two emotional states.

	Otuput class		
Target class	Emotions	Happy	Sad
	**Happy**	**32 (100%)**	0 (0%)
	**Sad**	1 (3.125%)	**31 (96.875%)**

*Bold value indicates high percentage of accuracy*.

**Table 6:** Confusion matrix for three emotional states.

	Otuput class			
Target class	Emotions	Happy	Sad	Joy
	**Happy**	32 (**100%**)	0 (0%)	0 (0%)
	**Sad**	0 (0%)	30 (**93.75%**)	2 (6.25%)
	**Joy**	4 (12.5%)	0 (0%)	28 (**87.5%**)

*Bold value indicates high percentage of accuracy*.

**Table 7:** Confusion matrix for four emotional states.

	Otuput class				
Target class	Emotions	Happy	Sad	Terrible	Joy
	**Happy**	29 (90.625%)	0 (0%)	3 (9.375%)	0 (0%)
	**Sad**	0 (0%)	29 (90.625%)	2 (6.25%)	1 (3.125%)
	**Terrible**	1 (3.125%)	2 (6.25%)	29 (90.625%)	0 (0%)
	**Joy**	3 (9.375%)	0 (0%)	2 (6.25%)	27 (84.375%)

**Table 8:** Confusion matrix for five emotional states.

	Output class					
Target class	Emotions	Happy	Sad	Terrible	Joy	Mellow
	**Happy**	26 (81.25%)	0 (0%)	3 (9.375%)	0 (0%)	3 (9.375%)
	**Sad**	0 (0%)	29 (90.625%)	2 (6.25%)	0 (0%)	1 (3.125%)
	**Terrible**	1 (3.125%)	2 (6.25%)	28 (87.5%)	0 (0%)	1 (3.125%)
	**Joy**	2 (6.25%)	0 (0%)	2 (6.25%)	27 (84.375%)	1 (3.125%)
	**Mellow**	3 (9.375%)	0 (0%)	2 (6.25%)	1 (3.125%)	26 (81.25%)

## Discussion

Emotion recognition using EEG signals has received much attention recently. EEG is an invaluable source of information about the brain dynamic and is Inherently nonlinear and highly complex (Soroush et al., 2020). External or internal stimulation, such as eliciting emotions, causes brain activity to become more complex. Accordingly, in order to study these types of systems, the use of nonlinear time series descriptors like what is performed in this study is imperative. Given the Symbolic Time Series Analysis (STSA) potential capabilities for nonlinear analyzing the EEG dynamics, it was used to recognize the emotional states in this study. Moreover, it was shown that low computational complexity and noise robustness are the other advantages of this method (Daw et al., 2003). Most of the different approaches to STSA have been proposed in the one-dimensional signal space. A person’s emotions, however, are not localized in any particular area of their brain. Neural circuits responsible for emotion regulation are distributed along different brain regions (Soroush et al., 2020). Therefore, we have used the entire 32-channel EEG signal for the whole brain, and the partitioning of the phase space has been done based on our proposed Spherical Phase Space Partitioning. To achieve more information, we performed the analysis in the phase space of all 32 EEG channels (instead of applying the STSA directly on the EEG time series themselves) and partitioned the phase space based on our proposed Spherical Phase Space Partitioning. Applying to the DEAP dataset, our proposed method has successfully identified all four combinations of emotional states. The comparison with other methods applied on the same dataset (DEAP) or other datasets is shown in Tables s 1–4. The evaluation indicated that our proposed method can recognize emotions more accurately than several different methods (which are represented in bold in Tables). For instance, the emotion recognition system which has been proposed by Liu and Sourina (2013), based on Higher-Order Crossing (HOC) features and Support Vector Machine (SVM) classifier, for two emotional states of DEAP, had an accuracy of 90.35%. In contrast, our proposed method had the accuracy of 98.44% on the same number of emotional states of DEAP. Also, for three combinations of emotions, they had an accuracy of 84.41%, while our proposed method had an accuracy of 93.75%. Increasing the classification accuracy can be seen in two other cases for four and five combinations of emotional states.

For four emotional states, the confusion matrix in Table 7 indicates that the happy, sad and terrible were classified with 90.625% of accuracy, and joy was classified with 84.375%. This indicates that in this group, joy is the most difficult to classify. It may be due to the similarity of the brain’s behavioral patterns in joy to states such as happiness. As is seen, in 9.375% of cases, the state of joy is mistakenly classified as happiness, which can indicate the similarity of the dynamic pattern of the brain in the case of joy to happiness.

## Conclusion

This study proposed an emotion recognition system based on a new Symbolic Time Series Analysis approach. Using the DEAP dataset, the proposed EEG-based emotion recognition system has successfully identified the emotional states of happiness and sadness, with an accuracy of 98.44% in a subject-independent approach. By increasing the number of emotions, the accuracy rates of 93.75%, 89.06%, and 85% were obtained for three, four, and five groups of emotions, respectively. The key strength of the proposed method is that while the dynamic characteristics of the signals are preserved, it is also a simple and fast method, which has a significant advantage, especially in real-time applications. For our future work, a potential research direction worth considering would be reducing the number of channels based on the importance of the brain area in the emotional states, which some methods like ICA could do. In addition, since the DEAP dataset videos are more based on the dimensional definition for emotions and just a few numbers of videos are labeled based on the discrete definition, more studies on DEAP have attempted to classify the emotions based on the arousal and valence axes. Future works can be conducted to use our proposed method to classify the arousal and valence axes or four areas of the space.

## Data Availability Statement

The data analyzed in this study is subject to the following licenses/restrictions: In this study, the DEAP database has been used. This database is available on: https://www.eecs.qmul.ac.uk/mmv/datasets/deap/download.html, but to access it, its collectors must be requested to be issued a password and provided it to you. Requests to access these datasets should be directed to https://anaxagoras.eecs.qmul.ac.uk/request.php?dataset=DEAP.

## Author Contributions

AM and HT contributed to the conception and design of the study. AM is the supervisor and corresponding author. HT performed the analysis and wrote the first draft of the manuscript. All authors contributed to the article and approved the submitted version.

## Conflict of Interest

The authors declare that the research was conducted in the absence of any commercial or financial relationships that could be construed as a potential conflict of interest.

## Publisher’s Note

All claims expressed in this article are solely those of the authors and do not necessarily represent those of their affiliated organizations, or those of the publisher, the editors and the reviewers. Any product that may be evaluated in this article, or claim that may be made by its manufacturer, is not guaranteed or endorsed by the publisher.
